# HPLC-DAD-MS and Antioxidant Profile of Fractions from Amontillado Sherry Wine Obtained Using High-Speed Counter-Current Chromatography

**DOI:** 10.3390/foods10010131

**Published:** 2021-01-09

**Authors:** Mónica Schwarz, Fabian Weber, Enrique Durán-Guerrero, Remedios Castro, María del Carmen Rodríguez-Dodero, Maria Valme García-Moreno, Peter Winterhalter, Dominico Guillén-Sánchez

**Affiliations:** 1 “Salus Infirmorum” Faculty of Nursing, University of Cadiz, 11001 Cadiz, Spain; monica.schwarz@uca.es; 2 Nutrition and Bromatology Area, Faculty of Medicine, University of Cadiz, Plaza Falla, 9, 11003 Cadiz, Spain; 3 Institute of Nutritional and Food Sciences, Molecular Food Technology, University of Bonn, Endenicher Allee 19b, D-53115 Bonn, Germany; fabian.weber@uni-bonn.de; 4 Analytical Chemistry Department, Faculty of Sciences-IVAGRO, University of Cadiz, Agrifood Campus of International Excellence (ceiA3), Pol. Río San Pedro, s/n, Puerto Real, 11510 Cadiz, Spain; remedios.castro@uca.es (R.C.); maricarmen.dodero@uca.es (M.d.C.R.-D.); valme.garcia@uca.es (M.V.G.-M.); dominico.guillen@uca.es (D.G.-S.); 5 Institute of Food Chemistry, Technische Universität Braunschweig, Schleinitzstrasse 20, 38106 Braunschweig, Germany; p.winterhalter@tu-braunschweig.de

**Keywords:** HSCCC, sherry wine, Amontillado, phenolic compounds, antioxidant activity

## Abstract

In the present work, the polyphenolic profile of a complex matrix such as Amontillado sherry has been processed by means of high-speed counter-current chromatography (HSCCC) and characterized by HPLC-DAD-MS. An Amberlite XAD-7 column was used to obtain the wine extract, and three different biphasic solvent systems were applied for HSCCC separation: MTBE (methyl *tert*-butyl ether)/n-butanol/acetonitrile/water (1.1/3/1.1/5+0.1% trifluoroacetic acid), MTBE/n-butanol/acetonitrile/water (2/2/1/5), and hexane/ethyl acetate/ethanol/water (1/5/1/5). As a result, 42 phenolic compounds and furanic derivatives have been identified by means of HPLC-DAD-MS, with 11 of them being identified for the first time in Sherry wines: 3-feruloylquinic acid, isovanillin, ethyl vanillate, furoic acid, dihydro-*p*-coumaric acid, 6-O-feruloylglucose, ethyl gallate, hydroxytyrosol, methyl protocatechuate, homoveratric acid and veratraldehyde. In addition, the antioxidant capacity (ABTS) of the obtained fractions was determined, revealing higher values in those fractions in which compounds such as gallic acid, protocatechuic acid, protocatechualdehyde, *trans*-caftaric acid, syringic acid, isovanillin or tyrosol, among others, were present. This is the first time that HSCCC has been used to characterize the phenolic composition of Sherry wines.

## 1. Introduction

Sherry wines, i.e., Finos, Amontillados and Olorosos, are famous throughout the world. From a legal point of view, the denomination of Sherry wines should only be used for wines produced under the Denomination of Origin Jerez-Xérès-Sherry, in the Southwest of Spain. These are wines that come from the same grape variety Palomino Fino, and that, after being pressed to different degrees, undergo different aging conditions [1]. The different degree of pressing during winemaking already determines the composition of the initial must with regard to many of its constituents, especially polyphenolic compounds. It is known that polyphenols possess various biological activities that may vary according to their structure [2]. The high structural diversity and complexity of the polyphenolic fraction, however, make it difficult to separate individual compounds and to study them in more detail. As the structural diversity is even increasing during the subsequent wine ageing process, novel analytical approaches such as, e.g., the preparative all-liquid chromatographic technique of countercurrent chromatography are required to fractionate the complex polyphenolic mixture to ensure a subsequent structure elucidation by HPLC-MS analysis.

Finos are wines that age under reducing conditions caused by the layer of yeast, known as veil of *flor*, which protects them from environmental oxygen and gives rise to a lower alcohol content compared to Olorosos and Amontillados wines. Olorosos are aged in American oak barrels under oxidizing conditions and with an alcohol content of around 18% alcohol, while Amontillados go through a period of biological ageing and are then fortified to 17–18% alcohol and subjected to oxidative ageing. All of them spend a fixed period of time in the cask according to a dynamic system known as *criaderas y solera*.

As can be seen from the above description, Amontillado is a very peculiar wine, with a phase of biological ageing and another later phase of oxidative ageing. Its phenolic composition must therefore be intermediate between Fino and Oloroso wines. In 1986, Estrella et al. [3] identified different low molecular weight phenols and other compounds in Fino, Oloroso and Amontillado Sherry wines. Benzoic acids (gallic, protocatechuic, *p*-hydroxybenzoic, vanillic, syringic and gentisic), cinnamic acids (caffeic, *p*-hydroxycinnamic and ferulic), phenolic aldehydes (*p*-hydroxybenzaldehyde, *p*-vanillinic, syringaldehyde and protocatechualdehyde), and the coumarins esculetin and scopoletin were among the compounds identified by them. Later, in 1996, Guillén et al. [4], after developing the method of automatic extraction by SPE of polyphenols from sherry wines, proceeded to determine them by HPLC. Their results confirmed this intermediate composition of the Amontillado wines compared to Fino and Oloroso ones in terms of polyphenolic content. Thirteen benzoic and cinnamic acids together with some aldehydes (protocatechualdehyde, vanillin, syringaldehyde, *p*-hydroxybenzaldehyde) and flavanols (catechin) were found in Amontillado wines by Guillén et al., highlighting the content of gallic acid, *trans*-*p*-coutaric acid and protocatechuic acid [4]. Furthermore, in 2002, García-Moreno and García-Barroso studied the evolution of the phenolic compounds in the three most important types of Sherry wines. In addition to those already found in previous works, they identified hydroxymethylfurfural (HMF) and tyrosol in samples of Amontillado wines [5]. More recently, Ortega et al. [6] studied the evolution of Olorosos’ polyphenolic content during their oxidative aging at different temperatures and observed that fluctuating temperatures caused the wines to age more rapidly with a greater increment in polyphenolic content. Flavanols were the polyphenolic fractions that appeared in the Olorosos studied in the highest concentration, and the (+)-catechin and procyanidin B1 contents were the most important. Other compounds, such as syringaldehyde and vanillin, showed clear gains as ageing time was increased.

Regarding Finos, it seems that the most abundant polyphenols are benzoic acid derivatives derived from the barrels’ lignin and from the deamination of the nitrogen compounds generated during the autolysis of the *flor* yeast [7]. It has been observed that with increasing ageing time the polyphenolic profile of Amontillado becomes closer to that of Oloroso than to Fino’s, with some aldehydes such as vanillin and *p*-hydroxybenzaldehyde increasing along with ageing time [5]. In this sense, López de Lerma et al. [8] failed to use an electronic nose to differentiate Amontillados from Olorosos, although the differentiation was complete with respect to Finos, both for the young and the sweet varieties.

As it can be seen from the scarce background presented above, the polyphenolic profile of this particular Sherry wine, Amontillado, has been barely studied until now. Therefore, in this work, we intend to develop a simple and effective method to isolate and identify simultaneously, and at a preparative scale, the different polyphenolic compounds present in Amontillado wine while considering the requirements for quantitative analysis of the bioactive compounds. For this purpose, we are proposing the use of high-speed counter-current chromatography (HSCCC) together with off-line analysis by HPLC-ESI-MS. Counter-current chromatography is a chromatographic technique that separates solutes by their different distribution coefficients between two immiscible solvents. This type of chromatography presents multiple advantages over other techniques. Among these, we would like to mention the following: high efficiency to separate and isolate bioactive natural products in short elution periods [9]; high recovery of injected samples, as no chromatographic column is required and, hence, irreversible adsorption does not occur [10]; high versatility of the application range; and high repeatability. In addition to these advantages, the simpler polyphenolic fractions that can be obtained allow us to evaluate their antioxidant activity and relate them with the identified polyphenols. This is a frequent application of HSCCC [11].

HSCCC has been widely used for separating bioactive compounds in numerous food matrices [12,13,14]. In the oenological field, there are also many studies that have used HSCCC. Thus, it has been successfully applied to the fractioning and separation of polyphenols in red wine [15]. Seventeen polyphenolic compounds including phenolic acids, catechins, proanthocyanidins and anthocyanins have been separated with high purity levels and large yields. In addition, Weber and Winterhalter [16], isolated and identified different anthocyanin oligomers by HSCCC and NMR, while Noguer et al. [17] investigated the antioxidant properties of different fractions obtained by HSCCC. This technique has also been successfully applied to identify and isolate polyphenolic compounds in grape skins and seeds [18], white wine [19,20] and rum [21], among other matrices.

The main objective of the present work is to characterize the polyphenolic compounds and furanic derivatives in a complex matrix such as Amontillado sherry by obtaining simpler fractions through HSCCC, and also by evaluating their antioxidant activity. To date, this is the first time that this technique has been applied to Sherry wine.

## 2. Materials and Methods

### 2.1. Reagents

For the preparation of the extracts for HSCCC separation, the following analytical grade solvents were used: methanol, ethyl acetate, ethyl methyl ketone, MTBE (methyl *tert*-butyl ether), n-butanol, acetonitrile, and ethanol, which were acidified with either acetic acid, formic acid, or trifluoroacetic acid, all of which were purchased from Merck (Darmstadt, Germany).

For the HPLC–DAD–MS analyses, HPLC gradient grade methanol, acetic acid and water (Merck) were used. De-ionized water was purified by means of a Milli-Q system provided by Millipore (Bedford, MA, USA). The reference standards were purchased from Fluka (Buchs, Switzerland), Merck (Darmstadt, Germany), and Sigma (St. Louis, MO, USA).

To determine the extracts’ antioxidant activity, a saturated solution of Zn(CH_3_COO)_2_ (Panreac, Barcelona, Spain) and a solution of 2,2′-azino-bis(3-ethylbenzthiazoline-6-sulfonic acid) (ABTS) (Sigma– Aldrich, Madrid, Spain) were mixed in phosphate buffer medium (pH 6). 6-hydroxy-2,5,7,8-tetramethylchroman-2-carboxylic acid (Trolox) supplied by Sigma–Aldrich was used for calibration.

### 2.2. Wine Samples

Samples of Amontillado wine (21.5% alcohol by volume), VORS (Vinum Optimum Rare Signatum) category with 30 years of mean ageing, supplied by González Byass S.A. winery (Jerez de la Frontera, Spain) were used for this research.

### 2.3. Preparation of the Extracts

Nine liters of Amontillado wine diluted in water (1:4) to minimize the effect of its high alcoholic degree were loaded onto an Amberlite XAD-7 column (100 cm × 7 cm) to eliminate proteins, organic acids, residual sugars, and minerals. The flow rate was approx. 10 mL/min. The Amberlite column was pre-conditioned with 2 L of water. The diluted sample was washed using 3 L of water and subsequently eluted with 2 L methanol acidified with acetic acid (19:1 *v/v*). The extract was concentrated with a rotary evaporator under vacuum and lyophilized to obtain a total of 11.2 g of XAD-7 extract.

In order to obtain less polar fractions, the XAD-7 extract was extracted with ethyl acetate by a liquid–liquid extraction. For that purpose, the lyophilized XAD-7 extract (2 g) was dissolved in 500 mL water and was extracted with ethyl acetate (1:1 *v/v*) in a separation funnel. The organic phase was removed and the extraction was repeated three times using 500 mL of ethyl acetate for each extraction. The process was carried out in duplicate and the pooled organic phases were concentrated by means of a rotary evaporator and lyophilized to obtain 900 mg of dry ethyl acetate extract. We were interested in less polar compounds, so we focussed on the ethyl acetate extract, and the water fraction was removed.

### 2.4. Selection of Two-Phases HSCCC Solvents Systems

Based on the literature data and preliminary experiments, different biphasic solvents systems were tested to perform the fractionation of the samples by HSCCC [22,23,24]. Preliminary experiments consisted on the visual estimation of the distribution of the extract within both phases (organic and aqueous). The intensity of color in both phases gave us an initial estimation of the suitability of the solvent systems. The systems tested were: hexane/ethyl acetate/methanol (1/1.2/1), MTBE (methyl *tert*-butyl ether)/n-butanol/acetonitrile/water (2/2/1/5), hexane/ethyl acetate/n-butanol/water (1/10/1/10), MTBE/n-butanol/acetonitrile/water (1/3/1/5), n-butanol/acetonitrile/water (4/1/5), MTBE/n-butanol/acetonitrile/water [1.1/3/1.1/5+0.1% trifluoroacetic acid (TFA)], MTBE/n-butanol/acetonitrile/water (1/3/1/5+0.1% TFA), MTBE/n-butanol/acetonitrile/water (0.5/3.5/1/5), MTBE/n-butanol/acetonitrile/water (1/4/1/5). For the ethyl acetate extract, the following solvent systems were tested: hexane/ethyl acetate/ethanol/water (1/5/1/5), ethyl acetate/ethanol/water (5/1/5), ethyl acetate/n-butanol/water (1/5/6), ethyl acetate/n-butanol/ethanol/water (30/6/10/50).

For this purpose, 5 mg from each extract were dissolved in 10 mL of the different systems tested at a 1:1 ratio of aqueous phase to organic phase [25]. Later, an aliquot from each phase was analyzed by liquid chromatography with diode array detection, and the signal was recorded at 280 nm in order to determine the one with the best partition coefficient.

### 2.5. Separation by Means of High-Speed Countercurrent Chromatography (HSCCC)

In the case of the XAD-7 extract, the solvent applied to HSCCC consisted on solvent system I: MTBE/n-butanol/acetonitrile/water (1.1/3/1.1/5+0.1% trifluoroacetic acid) and solvent system II: MTBE/n-butanol/acetonitrile/water (2/2/1/5). On the other hand, for the ethyl acetate extract, the following solvent system III was used: hexane/ethyl acetate/ethanol/water (1/5/1/5).

The HSCCC equipment used was a CCC-1000 by Pharma-Tech Research (Baltimore, MD, USA), together with a Biotronik HPLC BT3020 pump from Jasco (Pfungstadt, Germany). The separation was conducted at ambient temperature, at 850 rpm and at 3 mL min^−1^ flow rate, classical operation conditions for this type of CCC instrument. Based on the previous experience of the research group, the aqueous phase was used as mobile phase in the *head to tail* elution mode. The employment of *head to tail* mode with aqueous phase as mobile phase in HSCCC would be the equivalent of employing reversed phase elution mode in regular liquid chromatography. Previous studies have demonstrated this elution mode to be effective in the separation of polyphenols in wine matrixes [17]. A quantity of 800 mg of the extract was dissolved in 20 mL of a 1:1 mixture of the organic and aqueous phases and this was injected via a sample loop.

The separation was monitored at 280 nm, using a K-2501 detector (Knauer, Berlin, Germany). The fractions were collected using test tube racks and an LKB SuperFrac collector (Pharmacia, Bromma, Sweden). The thin layer chromatography (TLC) analyses of all the recovered HSCCC fractions were performed on 60 F254 Merck silica gel plates (Darmstadt, Germany), with an ethyl acetate/ethyl methyl ketone/formic acid/water elution system (4/3.5/1/1). After being developed, the detection was performed by UV detection and derivatization with *p*-anisaldehyde-sulfuric acid–glacial acetic acid reagent followed by heating up to 105 °C. The homogeneity of the final fractions was checked with the help of the chromatograms and the TLC of the different fractions, which were subsequently characterized.

### 2.6. Characterisation of the Fractions Separated by HSCCC

The resulting fractions were analyzed by HPLC-DAD-MS, using a Waters 2695 equipment coupled to a Waters 2996 photodiode detector and to a Waters micromass ZQ mass spectrometer fitted with electrospray interface (ESI). The column was a Phenomenex Gemini C18 (250 × 2.0 mm, 5 μm particle size). Elution system: A solvent (3% methanol, 2% acetic acid, and 95% water) and B solvent (93% methanol, 2% acetic acid, and 5% water) under gradient conditions: 0 min 100% A, 5 min 95% A, 30 min 50% A, 40 min 50% A, 50 min 100% B. The column was re-equilibrated before the following injection. Prior to their injection into the HPLC equipment, the samples were filtered through a 0.22 µm pore-size membrane (Millipore, Burlington, MA, USA). The injection volume was 20 μL and the flow rate 0.2 mL min^−1^. The following parameters were used for ESI-MS identification: positive and negative ionization modes with N_2_ as drying gas at a flow of 11 mL min^−1^, 250 °C drying temperature, 3500 V capillary voltage, and 15 V capillary exit. The m/z scanning range covered the 100–1000 uma interval. The injection volume was 20 μL.

### 2.7. Antioxidant Activity Determination

In order to determine the antioxidant activity of the fractions, 1 mg of each fraction was dissolved into 500 μL of methanol. The method used was a previously developed electrochemical method [26] consisting on electrochemical oxidation in a solution of ABTS (2,2-azinobis(3-ethylbenzthiazoline-6-sulphonic acid)) (50 M), to which the sample to be tested was added.

## 3. Results and Discussion

### 3.1. HSCCC Fractionation Using Different Solvent Systems

**Figure 1:**
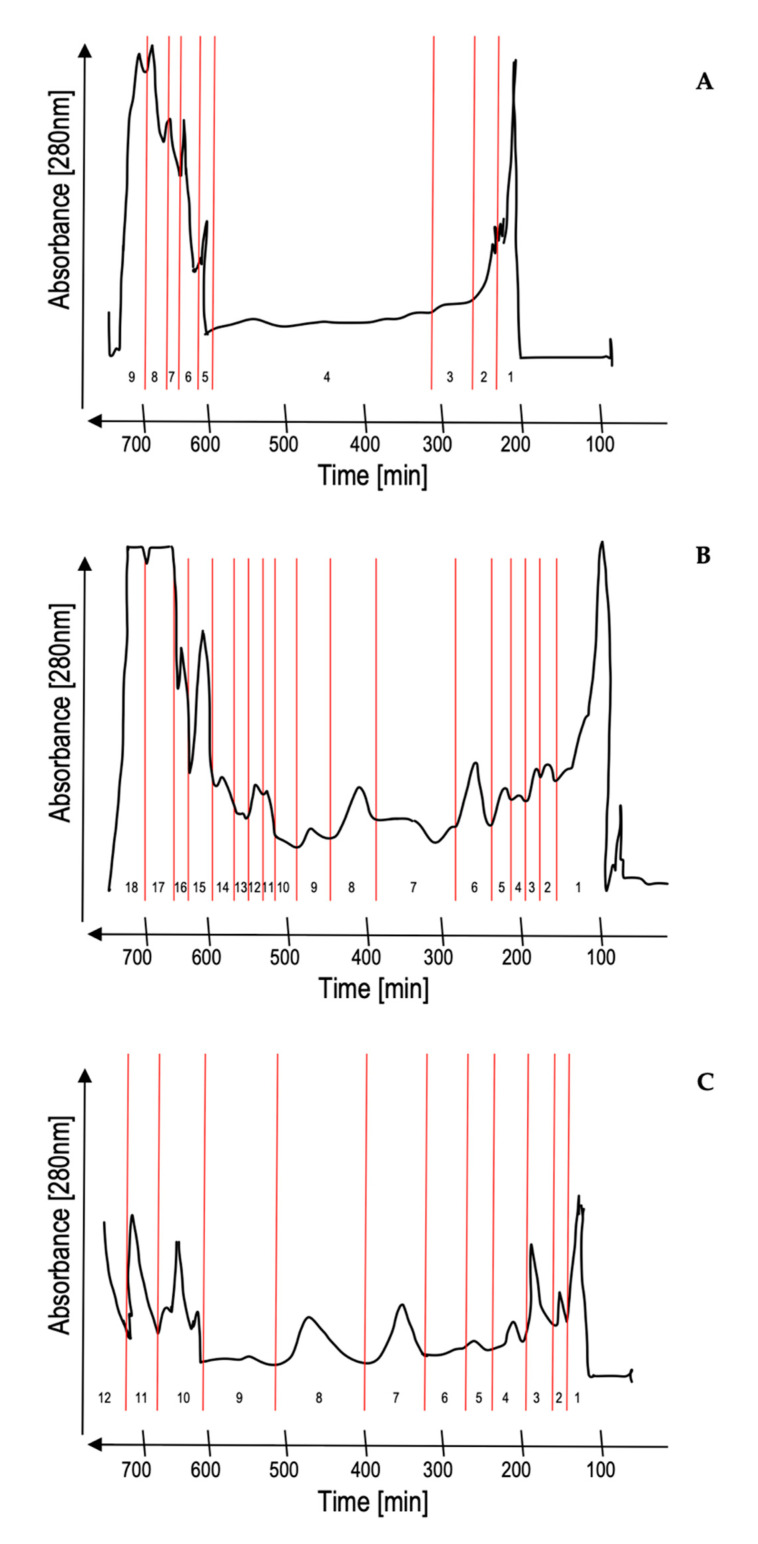
High-speed counter-current chromatography (HSCCC) fractionation obtained using the different solvent systems. (**A**)—solvent system I: MTBE (methyl *tert*-butyl ether)/n-butanol/acetonitrile/water (1.1/3/1.1/5+0.1% trifluoroacetic acid); (**B**)—solvent system II: MTBE/n-butanol/acetonitrile/water (2/2/1/5); (**C**)—solvent system III: hexane/ethyl acetate/methanol/water (1/5/1/5).

XAD-7 Amontillado extract (800 mg) was fractionated by HSCCC using the biphasic solvent system I, which consists on: MTBE/n-butanol/acetonitrile/water (1.1/3/1.1/5 *v/v/v/v*) acidified with 0.1% trifluoroacetic acid. Nine fractions were obtained, where fractions 6–9 were coil-fractions. In order to obtain simpler fractions, another solvent system was used. The second fractionation was carried out using the following solvent system II: MTBE/n-butanol/acetonitrile/water (2/2/1/5 *v/v/v/v*). After 12 h, the separation of 800 mg of XAD-7 Amontillado extract produced ten fractions in the normal elution mode and eight additional fractions in the extrusion mode. Upon injection of the solutes, the first stage in elution/extrusion CCC is a classical elution. In order to recover the compounds with a high affinity to the stationary phase, the extrusion mode is initiated by switching the solvent reservoir of the liquid pump system from mobile phase to stationary phase, i.e., making the formerly stationary phase the mobile liquid. As a consequence, the solutes located inside the column are subsequently pushed out of the column. The advantage of the elution/extrusion technique is that solutes cannot stay trapped in the column, i.e., a full sample recovery is achieved during an acceptable separation time [27]. The resulting fractions were simpler, but the coil-fractions still contained too many compounds. In order to simplify these fractions, the XAD-7 extract was re-extracted using ethyl acetate. A quantity of 700 mg of extract was employed and fractionated using solvent system III: hexane/ethyl acetate/methanol/water (1/5/1/5 *v/v/v/v*), and 12 fractions were obtained. Figure 1 shows the HSCCC fractionations obtained using the different solvent systems.

The quantities obtained in the three different fractionations after being concentrated and freeze-dried are shown in Table 1. The recovery of the sample from the three different fractionations was 50, 57 and 53%, respectively. The retained material was discharged and only the recovered sample was analyzed.

### 3.2. Identification of the Compounds in the Different Fractions Obtained by HSCCC

By analyzing the different fractions obtained by the HSCCC technique through HPLC-PDA-MS, a total of 42 phenolic compounds and furan derivatives were identified. The guidelines that were followed to carry out the identification of the phenolic compounds in the different fractions consisted of the acquisition of the UV-Vis and ESI-MS spectra, both in positive and negative mode, in order to ensure an identification of the molecular peak. Wherever reference compounds were available, the identification was confirmed by injecting the standard compound. In these cases, in addition to the corresponding MS and UV spectra, the retention time was also employed for the identification. For the identification of the remaining compounds, identification was carried out based on the mass spectrum, the UV-Vis spectrum, and the bibliographic data collected from the databases that are accessible on the Internet, such as Phenol-Explorer (Rothwell JA, 2015), PubChem, (PubChem, 2020), SpectraBaseTM (John Wiley & Sons, Inc., 2020), and ChemSpider (Royal Society of Chemistry, 2020). Table 2 shows the identifications that have been completed.

**Figure 2:**
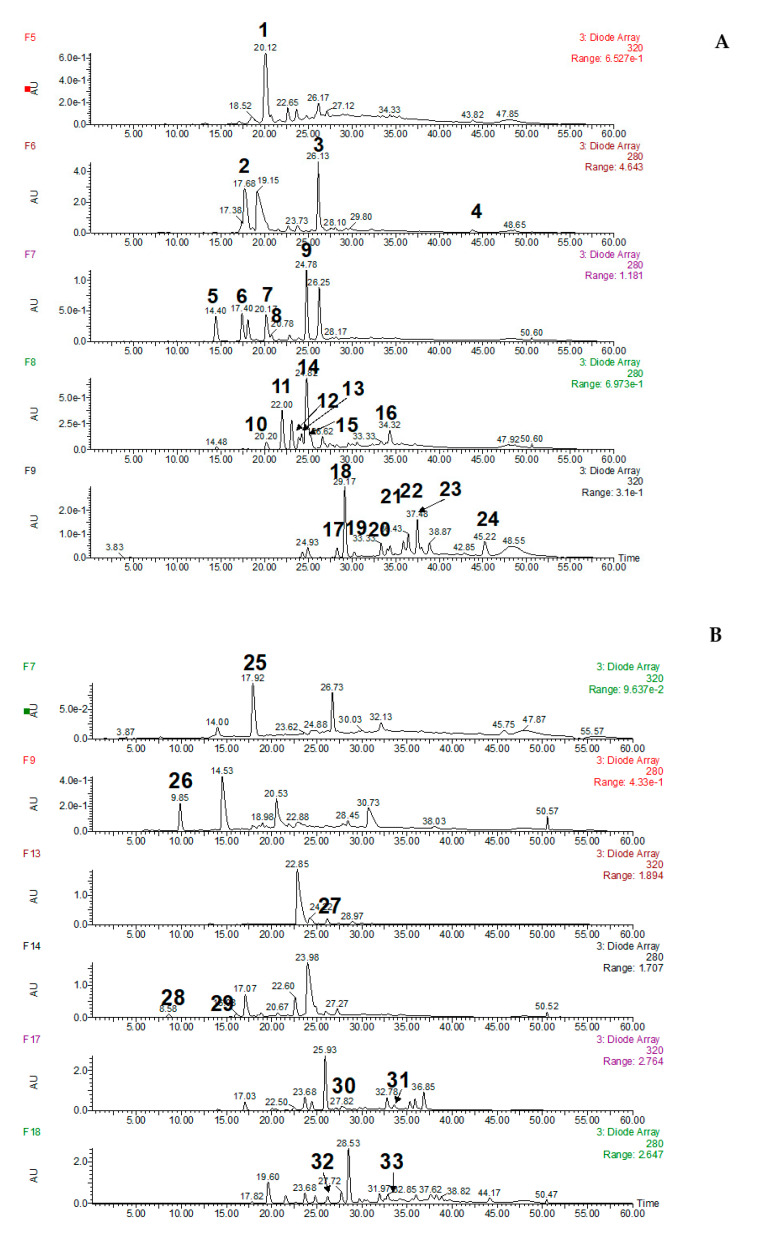

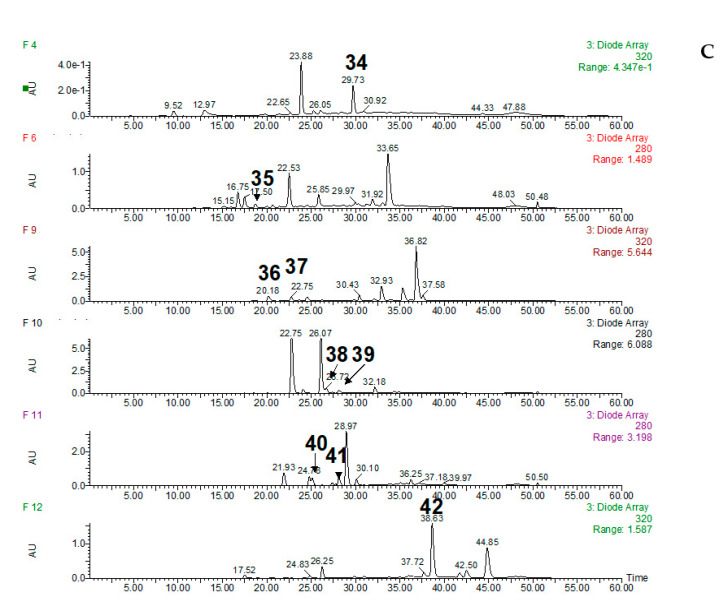
HPLC Chromatograms of the fractions obtained using the different solvent systems in which any compound was identified. The numbering applied corresponds to that used in Table 2. (**A**)—solvent system I: MTBE/n-butanol/acetonitrile/water (1.1/3/1.1/5+0.1% trifluoroacetic acid); (**B**)—solvent system II: MTBE/n-butanol/acetonitrile/water (2/2/1/5); (**C**)—solvent system III: hexane/ethyl acetate/methanol/water (1/5/1/5).

All the compounds identified, obtained with the different solvent systems, are reported in Figure 2. Only the chromatograms of the fractions in which any compound was identified have been presented. In the rest of the fractions, a very scarce number of compounds was present and no identification was carried out.

By comparing the MS and UV spectra and retention time of reference compounds, where available, 21 phenolic compounds and furan derivatives were successfully identified (Table 2). Thus, different hydroxybenzoic acids were identified, such as gallic acid (N.28), protocatechuic acid (N.5) and *p*-hydroxybenzoic acid (N.10); some methoxybenzoic acids as syringic acid (N.9), vanillic acid (N.12) and veratric acid (N.33); hydroxycinamic acids, such as caffeic acid (N.14), *trans-p*-coumaric acid (N.18) and *trans*-ferulic acid (N.19); esters of caffeic acid with tartaric acid (*cis*-caftaric acid (N.1) and *trans*-caftaric acid (N.7)); some hydroxybenzaldehydes, such as protocatechuic aldehyde (N.6) and *p*-hydroxybenzaldehyde (N.11); methoxybenzaldehydes, such as syringaldehyde (N.3), vanillin (N.40) and veratraldehyde (N.41); some hydroxycoumarins like esculetin (N.37) and scopoletin (N.39); and finally tyrosol (2) and 5-hydroxymethylfurfural (N.26).

On the other hand, through the use of the different databases previously mentioned as well as literature references, we found several hydroxycinnamic acids and hydroxycinnamic acid derivatives (Table 2), since they all have the similar and characteristic UV spectra of these compounds, with a maximum wavelength between 310 and 330 nm.

Thus, *cis-p*-coumaric acid (N.17) was identified with a mass spectrum identical to *trans-p*-coumaric acid (N.18) in both positive and negative modes. However, in the UV spectrum a hypsochromic shift of the absorption band of about 15 nm was observed (which was confirmed by querying PubChem) as well as a longer retention time of the *cis* isomer. Similarly, this behavior was observed in the compound identified as *cis*-ferulic acid (N.24) with respect to the *trans* isomer (N.20). We also encountered the corresponding esters of the two isomers of *p*-coumaric acid with tartaric acid (*cis-p*-coutaric acid (N.13) and *trans-p-*coutaric acid (N.15), Table 2), both with a fragment at [M − H]^−^ 296 Da, which would indicate the molecular mass of the corresponding ester and have UV spectra similar to those of the hydroxycinnamic acids from which they originate. In the same way, we found the compound N.27, with the UV spectrum characteristic of hydroxycinnamic acids and with fragments in the mass spectrum pointing to a molecular mass of 326 Da, that corresponds to that of fertaric acid. In the mass spectrum, fragments [M − H]^−^ to 193 Da corresponding to ferulic acid are present, which would confirm the loss of the tartaroyl radical.

The compounds labeled as N.21 and N.22 (Table 2) exhibit a fragment [M − H]^−^ at 324 Da in their mass spectra, which would point to a possible molecular mass of 326 Da, and both present the fragment corresponding to caffeic acid (179 Da). Therefore, they have been identified as derivatives of caffeic acid 1 and 2, since we have no other data that would allow us to clarify their identification.

Compound N.4 was identified as the ester with the quinic acid of ferulic acid, as it presents a UV spectrum similar to that of ferulic acid, within the mass spectrum of fragments corresponding to the same. In addition, a peak that could be assigned to the loss of quinic acid (−194 Da) was observed.

Based on the UV and MS spectra obtained for compounds N.16, N.20, N.23, N.31 and N.34 (Table 2) we can conclude that all of them are derivatives of hydroxycinnamic acid based on their UV absorption spectrum, with spectra related to caffeic acid, *p*-coumaric acid and ferulic acid. Furthermore, besides the fragments of the corresponding acids, we found in the mass spectra a fragment corresponding to the loss of the remaining hexose moiety (−162 Da). Thus, in the mass spectrum of compound N.34, we found the fragment at m/z 163, which is characteristic of the derivatives of *p*-coumaric acid. After consulting the MassBank of North America (MoNA) mass spectral database (GLP License) by introducing the fragmentation of the mass spectrum, it could be observed that it coincides with the spectrum of coumaric acid O-glucoside. In this group of compounds, we found those labeled as N.16 and N.20 (Table 2). The mass spectra of both point at a molecular mass of 342 Da, both with the fragment corresponding to caffeic acid. They also showed the characteristic fragmentation that indicates the excision of the remaining hexose, suggesting that they are hexosides of caffeic acid. After consulting the mass spectra in MoNA spectra database, we identified compound N.16 as caffeic acid 4-O-glucoside and compound N.20 as caffeic acid C-hexoside. Compound N.23 was identified as coumaric acid O-hexoside based on its UV spectrum, which is similar to that of *p*-coumaric acid and its mass spectrum, which displays the characteristic fragments of *p*-coumaric acid (*m/z* 163) and the loss of the hexosyl radical (−162) that was confirmed in the consulted databases. The mass spectrum of compound N.31 reveals a molecular mass of 356 Da, with the characteristic fragments of ferulic acid and the loss of a glycosyl group. On the other hand, the UV spectrum also confirms the presence of a ferulic acid, which has been identified as 6-O-Feruloyl glucose (Table 2).

The compound N.30 (Table 2) presents UV absorption and mass spectra coinciding with those of dihydro-*p*-coumaric acid (phloretic acid), which is a hydroxyphenyl propanoic acid, according to the databases that have been consulted, and presents a molecular mass of 166 Da and a UV absorption band at 277 nm.

The compounds labeled as N.25, N.32 and N.36 have been identified as alkyl esters of 3 benzoic acids (Table 2). The compound N.25 is assigned to ethyl vanillate, having a [M − H]^−^ of 181 Da and a UV spectrum with two bands at 262 and 295 nm, and thus being the identification confirmed by the literature [21] and the consulted bases. In the same way, compound N.32 (ethyl gallate) is identified by [M − H]^−^ at 197 Da and a UV absorption band at 272 nm; these data have been confirmed in the literature [21] and in the consulted databases. Following the same guidelines, the compound N.36 was identified as methyl protocatechuate, presenting a [M − H]^−^ at 167 Da and a UV absorption band at 304 nm.

The compound N.42 presents in the mass spectrum a fragment [M − H]^−^ at 207 Da and a UV spectrum similar to that of caffeic acid with a maximum lambda at 326 nm, which indicates that it is a derivative of this acid, and after consulting the bibliography and the databases consulted in the present work, we concluded that it is ethyl caffeate.

A fragment [M − H]^−^ at 195 Da and a UV spectrum with a band at 279 nm are the characteristics of the compound N.38, which, after consulting the bibliography and the databases, was tentatively identified as homoveratric acid.

Finally, furoic acid, with a molecular mass of 112 Da and UV absorption band at 255 nm, was identified as compound N.29. Compound N.35 was identified as hydroxytyrosol, with a UV spectrum similar to tyrosol, with a maximum wavelength at 275 and a fragment [M − H]^−^ at 153 Da. Both were also confirmed via consultation of the databases used.

### 3.3. Prevalence of the Identified Phenolic and Furanic Compounds in Wines

According to the information available, some of the compounds mentioned in Table 2, have been identified for the first time in Amontillados or Sherry wines: 3-feruloylquinic acid (N.4), isovanillin (N.8), ethyl vanillate (N.25), furoic acid (N.29), dihydro-*p*-coumaric acid (phloretic acid) (N.30), 6-O-feruloylglucose (N.31), ethyl gallate (N.32), hydroxytyrosol (N.35), methyl protocatechuate (N.36), homoveratric acid (N.38) and veratraldehyde (N.41).

The rest of the compounds identified in the samples have been widely reported in the literature regarding the phenolic content of Amontillado Sherry wines. As it has already been indicated in our introduction, previous studies [3,4,5] demonstrated the presence of gallic, caffeic, *p*-coumaric (*cis* and *trans*), *p*-hydroxybenzoic, protocatechuic, syringic, vanillic, and ferulic acids; the aldehydes *p*-hydroxybenzaldehyde, syringaldehyde, protocatechualdehyde and vanillin; the phenolic acid esters caftaric acid, chlorogenic acid and *p*-coutaric acid (*cis* and *trans*); and flavan-3-ol (+)-catechin [4], tyrosol and HMF in Sherry wines. Other publications on the phenolic compounds content in Sherry wines, which are not necessarily Amontillado wines, indicate the presence of other compounds such as ethyl caffeate (N.42), fertaric acid (N.27), *cis*-ferulic acid (N.24), *trans*-ferulic acid (N.19) and furoic acid (N.29) [6,28,29,30,31,32], all of which have also been identified in our wine (Table 2).

The origins of these compounds, in Amontillado wine in particular and in fortified wines in general, are quite different. Some of them are derived from the raw material itself, others are formed during the fermentation processes (alcoholic and malolactic fermentation), and/or they may also come from the extraction phenomena that take place during the final ageing process in wooden barrels.

Gallic acid (N.28, Table 2) can be generated both from the starting grapes and from the hydrolysis of gallotannins in the barrels’ wood, especially during the first years of ageing [33]. The esters from phenolic acids such as fertaric acid (N.27), the *cis* and *trans* isomers from caftaric acid (N.1 and N.7), and the *cis* and *trans* isomers from *p*-coutaric acid (N.13 and N.15) (Table 2), come from the grapes themselves and their initial concentration decreases in the first stages of wine ageing (biological ageing stage) while their concentration levels remain the same once the wine enters the oxidative stage [5]. Other esters that have been identified in this work are ethyl caffeate (N.42), ethyl gallate (N.32) and ethyl vanillate (N.25) (Table 2). These compounds have not been detected in fortified wines, although they have been identified in some samples of Riesling grapes and in single-varietal wines [20].

Some aldehydes such as vanillin, syringaldehyde, sinapaldehyde and coniferaldehyde are derived from the decomposition of lignin during the wood toasting process [34]. The oxidation of these aldehydes will eventually give rise to their corresponding acids: vanillic, syringic, ferulic and coniferylic. Of all these compounds, we have identified six in our wine (Table 2): vanillic acid (N.12), syringic acid (N.9), *cis* and *trans* ferulic acid (N.24 and N.19), vanillin (N.40) and syringaldehyde (N.3). The limited presence of aldehyde-type compounds in the samples of Amontillado wine is due to the inhibition of these compounds by the *flor* yeast during its initial biological ageing stage [5]. However, the presence of *p*-hydroxybenzaldehyde (N.11) is characteristic of Sherry wines and vinegars because of their long ageing periods in oak barrels.

The presence of furans is associated with the toasting of the wood, and more specifically with the presence of hydroxymethylfurfural (N.26), which is related to the thermal degradation of the glucose in wood cellulose. We have not found in the literature any author reporting the presence of furoic acid (N.29) in samples of Amontillado or other Sherry wines, but we have found some references indicating the presence of this compound in aged Madeira wines [35] and in barrel-aged spirits such as whisky, brandy or rum [36].

The presence of tyrosol (N.2) in samples of Amontillado wines, and other fortified wines, has also been described by other authors [5,6,29,32], but not that of hydroxytyrosol (N.35) (derived from tyrosol via the hydroxylation of its aromatic ring), even though small amounts (between 1 and 5 mg/l) have been detected in red and white wines [37,38]. These two compounds can be considered as secondary metabolites of the tyrosine formed by the yeast during the alcoholic fermentation [39].

The identified coumarins (Table 2), scopoletin (N.39) and esculetin (N.37), as well as other coumarins identified in wines and distillates, are secondary metabolites that result from an intramolecular esterification of orthohydroxycinnamic acid forming lactones. These two compounds had already been detected in Sherry wine samples (Finos, Olorosos and Amontillados) by Estrella et al. in 1986 [3].

Dihydro-*p*-coumaric acid (N.30) is derived from the reduction of *p*-coumaric acid by lactic acid bacteria during the malolactic fermentation [40]. Isovanillin (N.8) [41] and veratraldehyde (N.41) [42] have been identified in oak-aged vinegar samples.

We have not found any information in the literature on the presence of homoveratric acids (N.38) in grapes or derived products. Nevertheless, veratric acid and veratraldehyde have been found in Sherry wines [43]. Nor have any references been found to indicate the presence of 3-feruloylquinic acid (N.4), which is quite frequently found in coffee beans and in coffee itself [44].

### 3.4. Antioxidant Activity of the Fractions Obtained

In order to try and determine the relationship between antioxidant activity and phenolic composition, the antioxidant activity of the fractions obtained from XAD-7 extract, and also those from ethyl acetate extract, was determined. Table 3 shows the antioxidant activity as measured in the different fractions, as well as their standard deviation (*n* = 3).

It can be seen (Table 3), in reference to XAD-7 extract, that the F7 fraction was the one with the significant highest antioxidant activity (*p* < 0.05). Some of the polyphenols identified in this fraction were protocatechuic acid, protocatechualdehyde, *trans*-caftaric acid, syringic acid and isovanillin. Several of these compounds have been previously related to the antioxidant activity of different oenological products [45,46,47,48,49], and therefore the high antioxidant capacity of this fraction is justified. It is also worth mentioning that when comparing the antioxidant activity of the raw extract (2238 mM Trolox), surprisingly, it presents a similar value to that of some of the fractions obtained and listed in Table 3. This would confirm the hypothesis of the antagonistic effect that some polyphenols may present in terms of antioxidant activity, as argued by some authors [50,51].

In relation to the fractions obtained from the ethyl acetate extract, some of them, such as F3, F8 and F11, were found to have high values for antioxidant activity (Table 3). On the one hand, gallic acid and different esters of cinnamic acids can be found in the F3 fraction. The F8 fraction, on the other hand, contains two major compounds: protocatechuic acid and tyrosol. According to some studies, the presence of all these compounds in wines and derivatives is related to their antioxidant capacity [47,52,53]. Besides, some of the polyphenols that, according to these results, seem to contribute to a greater extent to the antioxidant activity, such as protocatechuic acid or tyrosol, have also been reported in this sense by previous works conducted on Sherry wines [54]. Furthermore, hydroxytyrosol was identified in the F6 fraction (Table 3), which although available in wine, is the main antioxidant in virgin and extra virgin olive oils [55], and has been attributed biological activity, and therefore may contribute to an increase in antioxidant activity.

All the above described results lead us to conclude that the prevalence of certain phenolic compounds in Amontillado wine represent a substantial contribution to the in vitro antioxidant activity of the different fractions obtained.

## 4. Conclusions

The use of the HSCCC technique has been successfully applied to a complex matrix such as Amontillado sherry. This technique facilitates the fractionation of samples and, therefore, the characterization and identification of compounds. In this way, the identification of 42 compounds has been achieved, 11 of which were identified for the first time in sherry wines. HSCCC has been used for the first time to investigate the composition of Sherry wines, and has proven to be useful for the characterization of this type of fortified wine. Therefore, it would be interesting to apply this technique to other types of Sherry wines in the future, in order to broaden the existing knowledge on this type of highly complex wine.

## Figures and Tables

**Table 1:** Weight measured for the different fractions obtained from the different HSCCC solvent systems.

System I		System II		System III	
Fractions	Weight (mg)	Fractions	Weight (mg)	Fractions	Weight (mg)
F1	56	F1	75.3	F1	30
F2	32	F2	20.5	F2	13
F3	30	F3	8	F3	33
F4	22	F4	7.1	F4	14
F5	56.6	F5	8.4	F5	20
F6	20.9	F6	8	F6	30
F7	34.2	F7	7.2	F7	12.3
F8	52	F8	9.2	F8	50
F9	101.6	F9	5.9	F9	80
Total	405.3	F10	12.2	F10	24
		F11	8.5	F11	12.9
		F12	19.2	F12	58.5
		F13	12.5	Total	377.7
		F14	27		
		F15	17.3		
		F16	8.6		
		F17	112.5		
		F18	36.9		
		Total	404.3		

**Table 2:** Identifications of the compounds detected in the different fractions obtained using the three solvent systems (S_I, S_II, S_III).

N.	Compounds	Fraction	RetentionTime (min)	UV Bands (nm)	Mass Weight	Molecular Formula	[M + H]^+^	[M − H]^−^
1	*cis*-Caftaric acid ^a^	F5 S_I	20.1	327	312.23	C13H12O9	231	179/229/311
2	Tyrosol ^a^	F6 S_I	17.7	274	138.16	C8H10O2	121	137
3	Syringaldehyde ^a^	F6 S_I	26.1	309	182.17	C9H10O4	183	181
4	3-Feruloylquinic acid ^b^	F6 S_I	43.9	332	368.30	C17H20O9	164/370	163/206/368
5	Protocatechuic acid ^a^	F7 S_I	14.4	260, 290	154.12	C7H6O4	153	155
6	Protocatechualdehyde ^a^	F7 S_I	17.4	280, 311	138.12	C7H6O3	139	137
7	*trans*-Caftaric acid ^a^	F7 S_I	20.2	328	312.23	C13H12O9		179/311
8	Isovanillin ^a^	F7 S_I	20.8	277, 310	152.15	C8H8O3		151
9	Syringic acid ^a^	F7 S_I	24.8	275	198.17	C9H10O5		197
10	*p*-Hydroxybenzoic acid ^a^	F8 S_I	20.2	255	138.12	C7H6O3		137
11	*p*-Hydroxybenzaldehyde ^a^	F8 S_I	22.0	284	122.12	C7H6O2		121
12	Vanillic acid ^a^	F8 S_I	23.1	260, 290	168.15	C8H8O4		167
13	*Cis-p-*Coutaric acid ^b^	F8 S_I	23.9	312	296.23	C13H12O8		150/163/295
14	Caffeic acid ^a^	F8 S_I	24.8	323	180.16	C9H8O4		135/179
15	*trans-p-*Coutaric acid ^b^	F8 S_I	24.9	313	296.23	C13H12O8		150/163/295
16	Caffeic acid-C-hexoside 1 ^b^	F8 S_I	34.3	330	342.30	C15H18O9		179/341
17	*cis-p-*Coumaric acid ^b^	F9 S_I	28.3	297	164.16	C9H8O3		119/ 163
18	*trans-p-*Coumaric acid ^a^	F9 S_I	29.2	310	164.16	C9H8O3		119/163
19	*trans-*Ferulic acid ^a^	F9 S_I	30.3	323	194.18	C10H10O4		194
20	Caffeic acid-C-hexoside 2 ^b^	F9 S_I	33.3	332	342.30	C15H18O9		179/341
21	Derivative Caffeic acid 1 ^b^	F9 S_I	35.8	313	326.00	C15H18O8		178/324
22	Derivative Caffeic acid 2 ^b^	F9 S_I	36.4	315	326.00	C15H18O8		178/324
23	Coumaroyl hexoside ^b^	F9 S_I	37.5	315	326.30	C15H18O8		162/178/325
24	*cis-*Ferulic acid ^b^	F9 S_I	45.2	310	194.18	C10H10O4		192/193
25	Ethyl vanillate ^b^	F7 S_II	17.9	281, 320	196.20	C10H12O4		123/195
26	Hydroxymethylfurfural ^a^	F9 S_II	9.9	284	126.11	C6H6O3	127	
27	Fertaric acid ^b^	F13 S_II	26.1	326	326.25	C14H14O9		193/325
28	Gallic acid ^a^	F14 S_II	8.6	271	170.12	C7H6O5	171	169
29	Furoic acid ^b^	F14 S_II	14.2	151	112.08	C5H4O3	113	111
30	Dihydro-p-coumaric acid ^b^	F17 S_II	27.8	278	166,17	C9H10O3	167	
31	6-O-Feruloylglucose ^b^	F17 S_II	33.5	307	356,32	C16H20O9		193/323/355
32	Ethyl gallate ^b^	F18 S_II	26.2	272	198.17	C9H10O5	199	197
33	Veratric acid ^a^	F18 S_II	32.9	260, 294	182.17	C9H10O4	183	181
34	4-O-beta-D-Glucosyl-4-coumaric acid ^b^	F4 S_III	29.7	290, 329	326.30	C15H18O8		163/246/325
35	Hydroxytyrosol ^b^	F6 S_III	18.2	275	154.16	C8H10O3		154
36	Methyl protocatechuate ^b^	F9 S_III	20.2	307	168.15	C8H8O4		145/167
37	Esculetin ^a^	F9 S_III	22.8	300, 346	178.14	C9H6O4	129/179	131/177
38	Homoveratric acid ^b^	F10 S_III	26.7	279	196.20	C10H12O4		156/195
39	Scopoletin ^a^	F10 S_III	28.3	300, 344	192.17	C10H8O4	194	192
40	Vanillin ^a^	F11 S_III	24.8	280, 310	152.15	C8H8O3	153	151
41	Veratraldehyde ^a^	F11 S_III	27.3	278, 315	166.17	C9H10O3	168	165
42	Ethyl caffeate ^b^	F12 S_III	38.6	326	208.21	C11H12O4		207

^a^: Identified based on bibliography and by injection of standards; ^b^: Identified based on specific bibliography and databases.

**Table 3:** Antioxidant activity (mM Trolox) and standard deviation (*n* = 3) of the fractions obtained from XAD-7 extract and ethyl acetate extract.

XAD-7 Extract			Ethyl Acetate Extract		
Fractions	Antioxidant Activity * (mM Trolox)	Standard Deviation	Fractions	Antioxidant Activity * (mM Trolox)	Standard Deviation
F1	0.000 ^a^	0.000	F1	2.712 ^bc^	0.740
F2	0.704 ^abc^	0.823	F2	2.768 ^bc^	1.041
F3	1.963 ^cd^	0.183	F3	6.680 ^d^	0.250
F4	2.250 ^d^	0.535	F4	2.214 ^b^	0.256
F5	0.000 ^a^	0.000	F5	2.720 ^bc^	0.336
F6	1.451 ^bcd^	0.469	F6	3.631 ^bc^	0.952
F7	4.320 ^e^	0.864	F7	2.749 ^bc^	0.183
F8	2.180 ^d^	0.333	F8	4.142 ^c^	0.536
F9	0.220 ^ab^	0.249	F9	2.790 ^bc^	0.205
			F10	2.918 ^bc^	0.048
			F11	3.846 ^c^	0.521
			F12	0.236 ^a^	0.239

*: Different letters in one column mean significant differences between the antioxidant activity of the fractions for each extract, according to Tukey’s test (*p* < 0.05).

## Data Availability

Data sharing not applicable.
